# Integrating health technology assessment and the right to health: a qualitative content analysis of procedural values in South African judicial decisions

**DOI:** 10.1093/heapol/czab132

**Published:** 2021-11-12

**Authors:** Michael J DiStefano, Safura Abdool Karim, Carleigh B Krubiner

**Affiliations:** Department of Health Policy and Management, Johns Hopkins Bloomberg School of Public Health, 615 N. Wolfe Street, Baltimore, MD 21205, USA; Berman Institute of Bioethics, 1809 Ashland Avenue, Baltimore, MD 21205, USA; SAMRC/WITS Centre for Health Economics and Decision Science (PRICELESS SA), Office 233, 2nd floor, Wits Education Campus, 27 St Andrews Road, Parktown, Johannesburg 2193, South Africa; Berman Institute of Bioethics, 1809 Ashland Avenue, Baltimore, MD 21205, USA; Center for Global Development, 2055 L St., Washington, DC 20036, USA

**Keywords:** Values, ethics, accountability, human rights, priority setting, healthcare

## Abstract

South Africa’s move towards implementing National Health Insurance includes a commitment to establish a health technology assessment (HTA) body to inform health priority-setting decisions. This study sought to analyse health rights cases in South Africa to inform the identification of country-specific procedural values related to health priority-setting and their implementation in a South African HTA body. The focus on health rights cases is motivated in part by the fact that case law can be an important source of insight into the values of a particular country. This focus is further motivated by a desire to mitigate the potential tension between a rights-based approach to healthcare access and national efforts to set health priorities. A qualitative content analysis of eight South African court cases related to the right to health was conducted. Cases were identified through a LexisNexis search and supplemented with expert judgement. Procedural values identified from the health priority-setting literature, including those comprising Accountability for Reasonableness (A4R), structured the thematic analysis. The importance of transparency and revision—two elements of A4R—is evident in our findings, suggesting that the courts can help to enforce elements of A4R. Yet our findings also indicate that A4R is likely to be insufficient for ensuring that HTA in South Africa meets the procedural demands of a constitutional rights-based approach to healthcare access. Accordingly, we also suggest that a South African HTA body ought to consider more demanding considerations related to transparency and revisions as well as explicit considerations related to inclusivity.

**Key messages**

- South Africa’s Constitution includes ‘the right to have access to health care services’. There is thus an important opportunity to explore how the potential priority-setting work of a health technology assessment body in South Africa may be integrated with this existing health rights framework to mutually support access to healthcare, rather than exist in tension with it.
- Accountability for Reasonableness is likely to be insufficient for ensuring that health technology assessment in South Africa meets the procedural demands of its rights-based approach to healthcare access.
- To date, the systematic and transparent analysis of case law to inform the work of health technology assessment bodies has been largely overlooked. Thus, an important contribution of the methodology described in this study is its potential application in national contexts other than South Africa, especially in countries where there is a constitutional basis for ensuring that all have access to healthcare or where judicialization of healthcare access occurs.

## Introduction

South Africa’s move towards implementing National Health Insurance (NHI), in pursuance of Universal Health Coverage (UHC), includes a commitment to establish a health technology assessment (HTA) body to inform priority-setting decisions about which drugs and healthcare services should be covered (DOH, 2017). HTA is the systematic evaluation of the effects and impact of healthcare interventions according to criteria that commonly include, but is not limited to, clinical effectiveness and cost-effectiveness (O’Rourke *et al.*, 2020). HTA can support the decision of whether to cover a new treatment or service (as part of a health benefits package) by determining its value, however defined, relative to an alternative intervention and the set of existing benefits provided by a healthcare system.

Social values or ‘judgments made on the basis of the moral or ethical values of a particular society’ (Clark *et al.*, 2012) play an important role in HTA (Littlejohns *et al.*, 2012). Social values are broadly divided into two types: (1) substantive values, which describe criteria used to assess the features and impact of particular interventions to inform whether or not they should be covered, and (2) procedural values describing how the broader approach to priority-setting decision-making should be undertaken (Clark *et al.*, 2012). This study focuses on the latter, analysing landmark health rights cases in South Africa to inform the identification of country-specific ‘procedural’ values related to health priority-setting and their potential implementation in a South African HTA body. This study complements work conducted by the South African Values and Ethics for Universal Health Coverage (SAVE-UHC, 2019) project that developed a ‘substantive’ ethics framework for HTA in South Africa and is part of a larger project to analyse health rights cases for both substantive and procedural values to inform HTA.^1^

The focus on health rights cases is motivated in part by the fact that case law can be an important source of insight into the social values of a particular country (Heintz *et al.*, 2015). This focus is further motivated by a desire to mitigate the potential tension between a rights-based approach to healthcare access and national efforts to set health priorities (Rumbold *et al.*, 2017; Wang, 2020). The right to health and its corollaries (e.g. the right to healthcare) are increasingly common in national constitutions (Kavanagh, 2016). These rights have contributed in some countries to the judicialization of healthcare access, or the process by which individuals or groups attempt to gain access to healthcare through litigation: on the one hand, judicialization of healthcare access may undermine ostensibly reasonable health priority-setting by national governments working to implement UHC (Norheim and Wilson, 2014; Dittrich *et al.*, 2016; Wang *et al.*, 2020), contribute to inefficient decision-making or budget distortions (Yamin and Parra-Vera, 2009; Biehl *et al.*, 2019; Ettelt, 2020; Wang *et al.*, 2020), or perpetuate and exacerbate inequities in healthcare access (Andia and Lamprea, 2019), thereby fuelling the concern that there may be an irreconcilable tension between the right to health and priority-setting efforts; on the other hand, judicialization may in some contexts promote equity in healthcare access (Biehl *et al.*, 2016; Andia and Lamprea, 2019) or function as an important corrective and accountability mechanism when national governments fall short of their mandate to make reasonable health priority-setting decisions (Gloppen, 2008; Biehl *et al.*, 2016; Dittrich *et al.*, 2016; Rumbold *et al.*, 2017; Syrett, 2018; Biehl *et al.*, 2019; Wang, 2020).

South Africa’s Constitution includes ‘the right to have access to health care services’. There is thus an important opportunity to explore how the potential priority-setting work of an HTA body in South Africa may be integrated with this existing health rights framework to mutually support NHI, rather than exist in tension with it. The remainder of this introduction provides a brief literature review of procedural values for health priority-setting that further motivates the aim of this study.

### Procedural values for health priority-setting

Accountability for Reasonableness (A4R) is the dominant account of procedural values in health priority-setting. Norman Daniels and James Sabin developed A4R as a means for achieving fair and legitimate priority-setting decisions independent of the substantive values chosen to inform those decisions. A4R was meant to side step the apparent difficulty of achieving consensus on substantive values that can guide decision-making by tackling the ostensibly easier problem of agreeing on the procedural values that will lead to fair and legitimate decisions (Daniels and Sabin, 1997; Daniels, 2000; 2016). Many have called for integrating A4R into HTA (Daniels *et al.*, 2015; Baltussen *et al.*, 2016; Daniels, 2016; Daniels and Van Der Wilt, 2016; Oortwijn and Klein, 2019), and HTA bodies such as the United Kingdom’s National Institute for Health and Care Excellence (NICE, 2008; 2021) and the Netherland’s Zorginstituut (ZIN, 2017) have indeed done so.

A4R requires (1) publicity or transparency about the reasons for a decision, (2) the use of reasons that fair-minded people can agree are relevant to the task of health priority-setting, (3) a process for revising decisions following appeals and (4) enforcement to ensure these first three conditions are met (Daniels, 2000). Only three of the A4R conditions are procedural values in the sense described above since the relevance condition sets a substantive requirement for priority-setting (Rid, 2009). To be sure, identifying relevant reasons that all can agree on will require certain procedures such as participatory processes (Gruskin and Daniels, 2008), but A4R does not clearly specify these.

Although A4R has been highly influential, many have suggested additional procedural values to ensure fair and legitimate priority-setting decisions. Clark *et al*. (2012), in developing a conceptual framework for social values in priority-setting, explicitly include ‘participation’, defined as involving a range of different people in the decision-making, as a key procedural value. Other critics of A4R have echoed the explicit need for participation in priority-setting processes (Friedman, 2008; Sabik and Lie, 2008a; Rid, 2009; Maluka, 2011). Many different modes of participation are possible. For instance, Pratt *et al*. distinguish nominal consultation, wherein members of the public merely provide feedback following priority-setting decisions, from partnership, wherein members of the public engage in shared decision-making at each stage of priority-setting (Pratt *et al.*, 2016). The quality of participation is also important. Calls for ‘qualitative equality’ (Pratt *et al.*, 2016) and ‘participatory parity’ (Blacksher, 2012) have focused on ensuring that participants have meaningful opportunities for effective participation in priority-setting processes. Relatedly, Gibson *et al*. (2005) argue for ‘empowerment’ as an important procedural value and discuss several practical considerations for mitigating power disparities between participants in priority-setting processes to ensure that all are able to participate effectively. Rand (2016) has argued that the value of ‘fair consideration’, or taking reasons seriously and giving them their due weight, is needed to ensure that participants’ contributions to the priority-setting process are not merely tokenistic. In a sense, fair consideration ‘empowers reasons’ (Rand, 2016, p. 113) in much the same way that Gibson *et al*. argue that participants ought to be empowered.

In addition to ‘participation’, several commentators have explicitly identified ‘impartiality’ as a procedural value (Emanuel, 2000; Tsuchiya *et al.*, 2005; Bond *et al.*, 2020). At a minimum, impartiality would require minimizing conflicts of interest for those participating in the priority-setting process (Emanuel, 2000; Tsuchiya *et al.*, 2005). Impartiality may also require that all participants in priority-setting processes have an equitable opportunity to be heard (Bond *et al.*, 2020). In this way, the procedural value of impartiality relates to calls for empowerment to achieve meaningful and effective participation. ‘Consistency’ has also been suggested as an important procedural value; a consistent HTA process is one where the same set of rules and protocols is used to assess each health intervention (Tsuchiya *et al.*, 2005). However, the importance of consistency has also been questioned, as some amount of flexibility in the process may be needed to adapt to changing values and health needs (Charlton, 2019; Bond *et al.*, 2020; DiStefano and Krubiner, 2020).

Given the range and depth of discussions around procedural values in the health priority-setting literature, A4R may not reflect the procedural value commitments of any particular country. First, A4R describes two ‘essential’ procedural values (i.e. transparency and appeals/revision), but not all countries should be expected to agree on which procedural values are essential. For example, Sabik and Lie (2008b) found that some countries may be quite comfortable with an expert-driven health priority-setting process, suggesting that public participation may be less important in those contexts. Moreover, procedural values like participation and empowerment may be relatively more important in countries with long histories of inequity and oppression. In general, some have questioned the foundational premise of A4R and argued that finding agreement at the individual level on procedural values to guide decision-making may not be easier than finding agreement on substantive values to do so (Wailoo and Anand, 2005; Sabik and Lie, 2008a; Ceva, 2012). Likewise, it is reasonable to suppose that countries vary in terms of which procedural values are most significant to them.

Additionally, A4R does not provide specific guidance for how countries ought to implement abstract procedural values, like transparency or appeals and revision, in specific HTA policies. While this vagueness can be seen as an advantage of the theory—by allowing countries to flexibly incorporate these procedural values in the design of their priority-setting processes (Sabik and Lie, 2008a)—there is a need for further guidance regarding how procedural values should be implemented through HTA policies in specific national contexts such as South Africa.

Finally, some have argued that courts can help to enforce A4R if the law supports or requires the implementation of A4R’s procedural values in government priority-setting processes (Syrett, 2011; Flood and Gross, 2014). This will be especially important if the courts in South Africa do not engage with the substantive merits of HTA decisions and focus instead on whether these decisions are procedurally legal, as has been the case in the United Kingdom (Syrett, 2011). It is currently an open question whether and to what extent South African courts will legally enforce the procedural values that constitute A4R or procedural values beyond these as well as specific considerations regarding the implementation of these values in the work of an HTA body.

For these reasons, this study sought to analyse landmark health rights cases in South Africa to inform the identification of country-specific procedural values related to health priority-setting and their implementation in a South African HTA body.

## Methods

### Case selection

**Figure 1.**
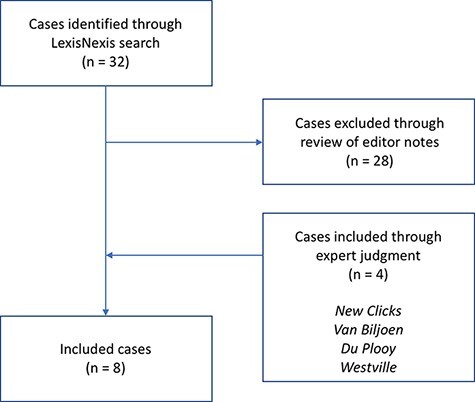
Case selection method.

As depicted in Figure 1, the case selection strategy combined the transparent and replicable approach common in the medical and social sciences with the use of expert judgement common in conventional legal scholarship. Case selection in legal scholarship has traditionally been informed by the authority and judgement of trained legal experts (Hall and Wright, 2008; Hall, 2013; Baude *et al.*, 2016), with little information provided to allow readers to assess the representativeness of cases (Baude *et al.*, 2016). While the conventional approach is likely suited to normative legal scholarship wherein researchers argue how the law ought to be interpreted, it is less appropriate when making descriptive claims about the law (Baude *et al.*, 2016), as the present study does. In the case of descriptive legal scholarship, transparent and replicable case selection can mitigate researcher bias and allow readers to better assess the accuracy and representativeness of claims (Baude *et al.*, 2016). For these reasons, Baude *et al*. (2016) have argued for a more transparent approach to conducting descriptive legal scholarship that adapts the approach commonly applied in the medical and social sciences.

This study focuses on South African judicial decisions related to the right to access healthcare and the State’s obligation to fulfil this right. Three sections of the South African constitution address the right to health: section 27 describes the socioeconomic right of everyone to have access to healthcare services, section 28 describes the right of children to basic healthcare services and section 35 describes the right of prisoners to medical treatment. We excluded section 28 from the sampling frame because the primary obligation to fulfil this right rests with parents and not the State (Government of the Republic of South Africa and Others v Grootboom and Others, 2000). The right to medical treatment in section 35(2)(e) was included because, in addition to addressing the rights of a vulnerable population, prisoners’ rights are considered a subset of section 27 rights (B and Others v Minister of Correctional Services and Others, 1997). The relevant text of each section is included in Table 1.

**Table 1.** Healthcare-related constitutional rights that confer a primary obligation of fulfilment on the South African state

27. **Healthcare, food, water and social security** (1) Everyone has the right to have access to— (a) health care services, including reproductive health care; (b) sufficient food and water; and (c) social security, including, if they are unable to support themselves and their dependants, appropriate social assistance. (2) The state must take reasonable legislative and other measures, within its available resources, to achieve the progressive realisation of each of these rights. (3) No one may be refused emergency medical treatment.
35. **Arrested, detained and accused persons** … (2) Everyone who is detained, including every sentenced prisoner, has the right— … (e) to conditions of detention that are consistent with human dignity, including at least exercise and the provision, at state expense, of adequate accommodation, nutrition, reading material and medical treatment…

We first conducted a search in September 2019 using Lexis Nexis. This search covered the years from 1996 to 2019 and was limited to South African Constitutional Law Reports. We used the following search strings to capture cases that included exact language from the health-related clauses of sections 27 and 35 of the Constitution: [27 AND ‘health care’ AND (‘reasonable legislative and other measures’ OR ‘available resources’ OR ‘progressive realisation’)] and [35 AND ‘medical treatment’ AND prisoner]. This resulted in 32 cases. One researcher reviewed the editor’s summary for each case and excluded cases for which the summary did not explicitly reference section 27(1), 27(2), 27(3) or 35(2)(e) as relevant to the case. Four cases were retained, all of which were section 27 cases. To achieve section 35 representation and to better ensure that no relevant case was overlooked, we supplemented these four cases with additional cases selected through expert judgement: one researcher with advanced academic and practical training in South African law (1) identified key reference texts (Cooper, 2011; Currie and De Waal, 2013; Bilchitz, 2014) that were cross-referenced to identify potential additional cases and (2) validated the selection of additional cases that resulted from this approach (more detail about the cross-referencing approach can be found in the Supplemental materials file). Through this approach, we selected four additional cases, resulting in a final sample of eight cases. Using the CiteIT signal in Lexis Nexis and the NoterUp section of Jutastat, we confirmed that none of these cases had been overturned as of September 2021. Table 2 provides summary details regarding the final sample, including the full title for each case (case abbreviations are used throughout the text).

**Table 2.** Case sample.

Case	Abbreviation	Year	Level of judgment	Case summary pertaining to section 27 or section 35 of the Constitution
Soobramoney v Minister of Health, KwaZulu-Natal (1997)	*Soobramoney*	1997	Constitutional Court	Soobramoney was in the final stages of chronic renal failure. Although he couldn’t be cured, his life could be prolonged through regular dialysis. At the time, the public healthcare system only provided dialysis for transplant candidates. After Soobramoney’s request for publicly funded treatment was denied, he brought a case arguing that the State was required to provide him with dialysis under his section 27 right to healthcare services. The Court found that the State hospital’s decision did not breach its obligations under section 27 due to the impact providing dialysis would have on healthcare system resources
Minister of Health and Others v Treatment Action Campaign and Others (2002)	*TAC*	2002	Constitutional Court	The government had created and implemented a pilot programme to interrupt mother-to-child transmission of HIV. This included administration of the drug, nevirapine, at the time of birth as well as additional services including provision of infant formula. The drug was only available in the private sector and two pilot sites in each province. TAC brought a case to compel government to provide nevirapine across the healthcare system, without the additional services, under section 27. The government argued that the nevirapine was not effective without the additional services and they did not have resources to expand the programme. The court found that the failure to provide nevirapine, without additional services, was unreasonable and fell short of section 27
Khosa v Minister of Social Development; Mahlaule v Minister of Social Development (2004)	*Khosa*	2004	Constitutional Court	Khosa, and the other applicants, were permanent residents of South Africa who had been denied State social security benefits. The Court had to determine whether the government’s decision to limit access to State social security benefits to citizens was compliant with section 27(2). Although the case does not concern the right to healthcare specifically, the right to access social security falls under the same section 27 and shares the same constitutional language and interpretation. The Court found that the restriction of benefits to citizens did not meet the standard of reasonableness under section 27
Minister of Health and Another v New Clicks SA (Pty) Ltd and Others (2006)	*New Clicks*	2005	Constitutional Court	The State had introduced amendments to the Medicines and Related Substances Act intended to make medicines more affordable. This was part of the State’s efforts to fulfil their section 27 obligation to provide everyone with access to healthcare services. The pharmaceutical and pharmacy industries opposed these measures, arguing in particular that the uniform medicine dispensing fee prescribed by the amendments would threaten the financial viability of pharmacies. The Court was divided on this issue. Six members found that the dispensing fee was inappropriate. The remaining five found that the dispensing fee was inappropriate only for rural and courier pharmacies
Mazibuko v City of Johannesburg (2010)	*Mazibuko*	2009	Constitutional Court	The City of Johannesburg introduced prepaid water meters in some areas. These meters dispensed 6 kl for free and thereafter shut off unless tokens were purchased. The previous system allowed consumers to use water and pay for water used at the end of the month. The case concerned whether the Free Basic Water policy, specifically the water shutting off after the 6 kl allowance, was a violation of section 27 of the Constitution (the rights to sufficient water and to access healthcare are both included under section 27). The Court found that the policy was constitutionally permissible
B and Others v Minister of Correctional Services and Others (1997)	*Van Biljoen*	1997	High Court	B and others were detainees in the South African prison system who were HIV positive and required antiretroviral treatments (ARVs). The question was whether the right to ‘adequate’ medical treatment for prisoners under section 35 gave them an entitlement to ARVs that they would have had access to through the public healthcare system outside prison. The applicants argued that section 35 required the State to provide them with this medically indicated therapy, even if it was not being provided at State expense in provincial hospitals. The Court found in favour of the applicants
Du Plooy v Minister of Correctional Services and Others (2004)	*Du Plooy*	2004	High Court	Du Plooy was a detainee in the South African prison system who was terminally ill and in need of palliative care. He sought release from prison on medical parole. The applicant’s request had previously been refused. Du Plooy argued for his release based on his constitutional rights to healthcare and medical treatment. The Court found that the decision not to place the applicant on medical parole violated sections 27 and 35 of the Constitution
E N and Others v Government of the Republic of South Africa and Others (2007)	*Westville*	2006	High Court	EN and others were prisoners at the Westville Correctional Centre who were HIV positive and were not given access to ARVs. They challenged the failure of the State to provide them with appropriate ARV treatment in fulfilment of sections 27 and 35 of the Constitution. The Court found in favour of the applicants and required the State to take steps towards the provision of appropriate ARV treatment as determined by the relevant medical authorities

Two of the cases identified for inclusion in the sample (*Mazibuko* and *Khosa*) address socioeconomic rights other than access to healthcare (i.e. the rights to sufficient water and social security, respectively). Because these rights are included under section 27, their interpretation by the courts is directly relevant to the interpretation of the right to access healthcare. To ensure a manageable sample size, we did not include cases that address socioeconomic rights enumerated under sections beyond section 27. However, and as can be seen in the results below, non-section 27 socioeconomic rights cases are sometimes quoted in the judgments of the included cases. We are therefore confident that our analysis still captures the relevant portions of any case judgments excluded from our sample.

### Analysis

The codebook for this analysis reflects procedural values drawn from the health priority-setting literature. To begin, we included transparency and appeals/revisions from A4R. We exclude A4R’s substantive condition—relevant reasons—from this analysis. In a separate study, we analyse the substantive values present in South African judicial decisions (DiStefano *et al.*, 2020). We also exclude enforcement as redundant since the aim of the study is to understand how the courts might enforce procedural values through their case judgments. We supplemented transparency and appeals/revision with an additional procedural value—inclusivity—drawn from work conducted by the Health Technology Assessment International Global Policy Forum (held in January of 2020) to identify core principles for deliberative processes in HTA. At this meeting, 80 HTA experts and practitioners from 22 countries engaged in various interactive activities to iteratively select core principles from a larger set previously identified in a literature review (Bond *et al.*, 2020). The group ultimately chose three principles: transparency, inclusivity and impartiality. Transparency was already included in our coding framework. Impartiality was described as involving considerations related to managing conflicts of interest and ensuring that all stakeholders are empowered to participate equitably. Given the close relationship between impartiality and considerations related to participation and empowerment, we grouped considerations related to impartiality under the theme of inclusivity.

Qualitative coding was conducted in MAXQDA 2020 (VERBI Software, 2019). Two researchers independently coded one section 27 case (*TAC*) and one section 35 case (*Van Biljoen*) to improve reliability in the application of codes and to identify additional codes inductively. Following this initial analysis, the two researchers discussed and resolved inconsistencies in the application of codes. These researchers then independently coded the remaining six cases in two batches, with further discussion and comparison following each batch. Analysis was limited to majority decisions or, in the one case where there was no majority decision (*New Clicks*), to the majority outcome.

Discussion between the two coders following the first round of coding led to the inclusion of an additional theme relating to which parties have the ability to bring claims before the courts (‘Individual vs collective claims’). This theme captures a procedural consideration of the courts themselves that might impact HTA with respect to health priority-setting. The full codebook is described in Table 3. The results below are organized by procedure and present relevant findings across all cases.

**Table 3.** Codebook.

Themes (values)	Sub-themes (related considerations)
Transparency (Daniels, 2000)	The reasons and rationales for a priority-setting decision are made public
Appeals/revision (Daniels, 2000)	Those impacted by priority-setting decisions should be able to formally appeal and there should be clear procedures revising decisions in the light of these challenges
Inclusivity (Bond *et al.*, 2020)	Appraisal committees should be appropriately representative There should be meaningful opportunities for participation by all relevant stakeholders Power differences among participants should be minimized The perspectives of participants are genuinely considered and responded to The chair or facilitator of deliberations manages discussions to ensure equitable input by all
Individual vs collective claims	Considerations regarding which parties have the ability to bring claims before the court

## Results

### Transparency

In *TAC*, the Court asserts that reasonableness in fulfilling the right to access healthcare demands transparency: ‘In order for it to be implemented optimally, a public health programme must be made known effectively to all concerned, down to the district nurse and patients. Indeed, for a public programme such as this to meet the constitutional requirement of reasonableness, its contents must be made known appropriately’.

In *Du Plooy*, the Court emphasized the importance of being transparent about the reasons that support policy decisions:

*According to the applicant…he was informed by the fifth respondent that his possible placement on medical parole was declined because he “…did not meet the criteria”. He was neither given any indication what these criteria were nor provided with the reasons for not being placed on medical parole. “The giving of reasons is one of the fundamentals of good administration.”*

The *New Clicks* judgment also highlights the necessity of transparency about reasons. The judgment points to a provision in the Constitution that requires every person to ‘be furnished with reasons in writing for administrative action which affects any of his or her rights or interests unless the reasons for such action have been made public’. The judgment adds that, ‘Transparency must be fostered by providing the public with timely, accessible and accurate information’.

The Court in *Mazibuko* writes, ‘A reasonableness challenge requires government to explain the choices it has made. To do so, it must provide the information it has considered and the process it has followed to determine its policy’. To explain why a policy is reasonable, the State ‘must disclose what it has done to formulate the policy: its investigation and research, the alternatives considered, and the reasons why the option underlying the policy was selected’. The reasonableness standard set by the Constitution therefore demands that the government be transparent about the reasons for and against its decision, as well as the broader decision-making process. The *Mazibuko* judgment also raises the concern that too much information could be overwhelming, writing that, ‘the applicants took issue with the sheer quantity of information placed before the courts by the City and Johannesburg Water in particular’.

### Appeals and revision

There were no findings specifically relating to appeals, although each case analysed in this case represents an appeal of a decision impacting the provision of healthcare. We further discuss the potential of the courts as a site for appeal below.

Regarding revision, the Court writes in *Mazibuko*: ‘The concept of progressive realisation recognises that policies formulated by the State will need to be reviewed and revised to ensure that the realisation of social and economic rights is progressively achieved’ and that ‘the obligation of progressive realization imposes a duty upon government continually to review its policies to ensure that the achievement of the right is progressively realized’. An important reason why the Court found that the Free Basic Water policy was reasonable was that the City had not ‘set its policy in stone’ and had instead ‘engaged in considerable research and continually refined its policies in the light of the findings of its research’.

### Inclusivity

The *New Clicks* judgment notes that, in public administration, ‘the public must be encouraged to participate in policy-making’. Additionally, the *New Clicks* judgment cites the constitution, which requires the National Assembly to ‘facilitate public involvement in the legislative and other processes of the Assembly and its committees’.

In *Mazibuko*, the Court notes that ‘all administrative decisions which affect the public must be preceded by public participation’ and describes the extensive opportunities for public participation prior to the implementation of the Free Basic Water policy: ‘…consultation processes were held through formal structures representing the community…[m]eetings and workshops were held with all 43 ward committees in Greater Soweto as well as public meetings’. In response to the applicants’ charge that more could have been done to involve the public, the Court writes, ‘[t]o require the City to provide notice and an opportunity to be heard each time a pre-paid allowance is about to expire, as the applicants contend, would be administratively unsustainable’.

### Individual vs collective claims

An important procedural legal matter addressed in *Westville* was whether the applicants were entitled to seek collective relief on behalf of all prisoners with HIV in their correctional facility. The Court found that they were. In support of this decision, the Court quoted an earlier judgment:

*It is precisely because so many in our country are in a “poor position to seek legal redress” and because the technicalities of legal procedure, including joinder, may unduly complicate the attainment of justice that both the interim Constitution and the Constitution created the express provision that “anyone” asserting a right in the Bill of Rights could litigate “as a member of, or in the interest of a group or class of persons.”*

Similarly, the applicants in *Khosa* claimed to act in the interests of others, not simply in their individual interests. Although the respondents challenged the applicants’ standing to make this claim, the Court found in favour of the applicants:

*…it is appropriate for the applicants to bring this matter in the interest of permanent residents and children who are in the care of permanent residents. They are indeed members of a group or class of people who would qualify for social assistance under the Act but for the fact that they are not South African citizens. They also act on behalf of children who cannot act on their own.*

## Discussion

The importance of transparency and revisions for the realization of the right to health in South Africa is evident in our findings, suggesting that the courts can help to enforce elements of A4R. While a South African HTA body could thus consider adopting A4R as a procedural values framework, A4R is likely to be insufficient for ensuring that HTA in South Africa meets the procedural demands of a rights-based approach to healthcare access. This is because, as discussed below, our findings suggest South Africa should consider adopting more demanding measures of transparency and revision than A4R calls for, as well as explicit approaches for encouraging and facilitating public inclusion in decision-making. These findings are consistent with, and extend, critiques of A4R in the health priority-setting literature.

### Transparency

Several of the judgments analysed in this study establish that the reasonableness standard in section 27 of the South African Constitution requires government transparency to some degree when making policy decisions affecting the right to access healthcare. Mansbridge (2009) has described two levels of transparency: transparency in rationale and transparency in process. Transparency in rationale means that the reasons or facts that directly support a decision are made public, while transparency in process means that all meetings, deliberation and research that led to a decision are made public regardless of whether they directly support the decision. This distinction has been adopted by some in the political science literature (De Fine Licht *et al.*, 2014,a,b), but it has not yet been factored explicitly into discussions in the health priority-setting literature. As discussed earlier, A4R calls for transparency in rationale. Both *Du Plooy* and *New Clicks* assert that the government must report its reasons for policy measures that impact healthcare access. According to these cases, then, the demands of a rights-based approach to healthcare access in South Africa are aligned with A4R’s requirement of transparency in rationale. In addition, the *Mazibuko* judgment asserts that the reasonableness standard requires the government to transparently report its broader research and decision-making processes, as well as reasons both for and against its ultimate decision. This judgement therefore calls for a degree of transparency that is closer to full transparency in process, a more demanding standard than A4R’s requirement of transparency in rationale.

Requiring transparency in process may have certain drawbacks (Mansbridge, 2009). For example, and as identified in the *Mazibuko* judgment, there is a risk of overwhelming the public if the focus of reporting is simply on making more information about the decision-making process available (O’Neill, 2002). This may be especially likely to occur if the government does not facilitate two-way communication with the public about the disclosed information (O’Neill, 2004) or if the health priority-setting approach adopted in South Africa is highly technical, such as those that rely largely on quantitative multi-criteria decision analysis (DiStefano and Krubiner, 2020). In response to concerns like these, some have argued that transparency should be understood as requiring active dissemination of relevant information through various media in a manner that makes the information understandable to different groups, particularly those that are the most disadvantaged (Naurin, 2007; Rid, 2009; Persad, 2019). It is thus noteworthy that the *New Clicks* judgment asserts that transparency requires ‘accessible’ information; fostering accessibility may require the active dissemination and engagement envisioned by the critics of traditional notions of transparency. There may yet be other drawbacks associated with implementing transparency in process, especially in its most extreme forms, such as incentivizing public posturing at the expense of high-quality decision-making (Naurin, 2003; Chambers, 2004; Mansbridge, 2009). Going forward, policymakers in South Africa ought to carefully consider how to mitigate these risks and address the trade-offs that can arise when implementing demanding transparency requirements for health priority-setting in HTA.

### Appeals and revision

There were no findings specifically related to appeals processes, although of course the courts offer one route for public appeals of health priority-setting decisions (each of the decisions in these cases was an appeal of an initial decision taken by government or by a healthcare provider). With this in mind, policymakers in South Africa should consider the potential benefits and costs of designing HTA to shoulder varying degrees of the burden of these appeals. For instance, a highly accessible HTA appeals process may help to lessen the courts’ burden, freeing them up to focus on other cases but could limit the ability of an HTA body to invest in other important processes such as participatory processes. The relative legitimacy of an HTA body vs the courts may also influence which institution ought to shoulder the burden of appeals. If an HTA body is perceived as more democratically accountable than the courts, it may be preferable to design HTA to accommodate the majority of appeals.

This study’s findings also suggest an alternative to the way of thinking about revision offered by A4R. A4R describes appeals and revision as a single condition (Daniels and Sabin, 1997; Daniels, 2000), implying that revisions ought to follow or be triggered by formal appeal processes and thus placing the burden of initiating revisions on those who formally appeal. In contrast, the *Mazibuko* decision establishes that the need for policy revision is inherent in the constitutional obligation to progressively realize socioeconomic rights like access to healthcare. This interpretation of the constitutional obligation to progressively realize socioeconomic rights both delinks the need for revisions from appeals processes and shifts the burden for initiating revisions from those impacted by the government’s decisions to the government itself. In the South African context, where the progressive realization of the right to access healthcare is required, whether revisions take place should not exclusively rely on whether a formal appeal of a decision is made. An HTA body in South Africa should therefore consider establishing procedures for conducting regular reviews of its coverage decisions that do not depend on the initiation or outcomes of appeals. Such regular reviews of decisions at the national level to include or exclude particular healthcare interventions from health benefits packages are not common (Glassman *et al.*, 2016). A South African HTA body thus has an opportunity to model this focus on revision. Additionally, establishing procedures for conducting regular reviews may reduce some of the bias that can result from a system wherein those impacted by an HTA decision initiate appeals given that those who do so almost always favour the technology’s adoption.

### Inclusivity

The *New Clicks* judgment asserts that the government must ‘encourage’ and ‘facilitate’ public involvement in its processes. This decision suggests that simply providing opportunities for public involvement in health priority-setting is insufficient. Pratt *et al*.’s (2016) theory of ‘deep inclusion’ is helpful for understanding why actively encouraging and facilitating public involvement in priority-setting may be critically important. According to Pratt *et al*., ‘deep inclusion’ requires careful consideration of both the ‘range’ and ‘mass’ of different perspectives when designing participatory processes. ‘Range’ refers to the types of people who are included in terms of both demographics and their role played in the health system (e.g. clinicians, researchers and patients), while ‘mass’ refers to the number of people who represent each category. Pratt *et al*. emphasize the importance of achieving a ‘critical mass of various perspectives’ and avoiding disproportionate representation by any one group. Many who could provide an important perspective may not be aware of the opportunities available to participate in health priority-setting at the national level or may not have the time and resources to take advantage of the opportunities even if they are aware. Active outreach to identify representatives of the groups and demographics most likely to be overlooked or under-represented, including groups that do not have a direct interest in the technology under consideration by an HTA body, can help to address these concerns and achieve ‘critical mass’ for different perspectives. Providing material support to patient groups who wish to submit evidence to inform priority-setting processes may also be necessary (Rozmovits *et al.*, 2018; Mercer *et al.*, 2020).

Pratt *et al*. (2016) also discuss the necessity of ensuring ‘qualitative equality’ among those who participate in priority-setting processes. Qualitative equality requires equal and effective opportunities to give one’s perspective and to question and respond to other participants, in addition to freedom from coercion or the pressure to accept or reject specific priority-setting proposals (Young, 2000; Pratt *et al.*, 2016). Achieving qualitative equality during deliberative appraisal sessions, decision-making or other stages of health priority-setting will likely require active facilitation to limit the influence of power disparities between participants and ensure that all participants are empowered to contribute. Gibson *et al*. (2005) have suggested a number of specific measures that may facilitate empowerment in priority-setting processes such as closed voting procedures and incorporating education and training for participants related to particular methods of evidence generation and appraisal. A committee chair may also play a critical role in facilitating qualitative equality among participants by mitigating the influence of dominant voices and encouraging all willing participants to speak often and openly (Krubiner and Ollendorf, 2019).

This finding that HTA in South Africa should explicitly commit to deliberately incorporating processes that encourage and facilitate public inclusion adds important content to A4R’s implicit and vague commitment to inclusive processes as a means of identifying relevant reasons. Of course, there will be costs and trade-offs associated with implementing more robust participatory procedures, as is noted in the *Mazibuko* judgment above. Future research should more carefully explore the trade-offs that stakeholders in South Africa are willing to make between implementing different participatory processes and the overall functioning of an HTA body given available resources.

### Individual vs collective claims

South African jurisprudence is notable for its focus on collective rights claims regarding access to healthcare. This approach differs from systems where judicialization of the right to health occurs largely through thousands of individual claims, as in Brazil (Ferraz, 2009; Syrett, 2018) and Colombia (Yamin and Parra-Vera, 2009). This is particularly important for HTA to consider since a focus on collective rights claims means courts could reverse decisions made by an HTA body for all potential beneficiaries in need of a health intervention that was previously excluded from the benefits package. This further underscores the importance of working to ensure that HTA procedures are integrated with the existing health rights framework.

Interestingly, our findings also suggest that the South African courts’ focus on collective claims is at least partially motivated by considerations of equity, one of the principles identified as underpinning the development of NHI in South Africa (DOH, 2017). The *Westville* judgment discussed above explicitly acknowledges that many people in South Africa will lack the resources or practical knowledge to pursue individual rights claims. Collective claims made on behalf of classes of similarly situated persons may thereby benefit those who would have been unable to make an individual appeal within the judicial system. The impact of judicialization on equity in healthcare access, however, is a complex and unsettled empirical question. Researchers disagree about whether the individualist approach in countries like Brazil and Colombia has entrenched inequities or has instead promoted fairer access to healthcare resources (Andia and Lamprea, 2019; Biehl *et al.*, 2019). Moreover, the impact of collective claims on equity is relatively understudied (Biehl *et al.*, 2019). In contexts like South Africa, there are not enough cases to support quantitative inferences about the impact of judicialization on equity in healthcare access. New research approaches are needed to validate the aspiration expressed in the *Westville* judgment through achieving a better understanding of the distributional impacts of judicialization on access to healthcare in South Africa.

### Limitations

One limitation of this study is the focus on majority judgments only. As the purpose of these studies is to provide policy recommendations to an HTA body, limiting analysis to only those judgments that currently constitute the law in South Africa should result in clearer and more practicable insights for policymakers. Additionally, the cases identified for analysis were not typically divisive; because only three of the eight cases included any concurring or dissenting opinion, the choice to exclude concurring and dissenting opinions from the analysis did not entail substantial information loss. To be sure, dissenting opinions may contribute over time to jurisprudence and constitutional interpretation, especially in South Africa, where constitutional values are considered culturally and socially contingent and may evolve over time (Mothupi, 2005). Dissenting opinions also ensure that differing perspectives are made public (Spies, 2020), regardless of their eventual influence on law. These differing perspectives may be important for informing HTA work even if they do not form the basis of law. Concurring opinions may of course have similar significance. Any future efforts in other countries to identify social values in case law should thus consider the merits of incorporating concurring and dissenting opinions in content analyses, especially in contexts where legal precedent is unstable or where there is typically greater disagreement among judges.

Importantly, insights from health rights case law can only partially establish the procedural values that ought to inform the work of an HTA body. One reason is because procedural values may be sufficiently generalized that important expressions or specifications of these values relevant to HTA may appear in non-health rights cases not already included in our sample. As such, our analysis represents a starting point for HTA in South Africa; key procedural values could also be identified and interpreted through the analysis of further case law, as well as national legislation, engagement with moral and political philosophy, and by surveying and entering into deliberations with the public and communities likely to be affected by HTA decisions. Moreover, further work to develop the procedural infrastructure of HTA in South Africa could consider how different procedures might reflect, advance or be traded off with the substantive value commitments of this HTA body. The SAVE-UHC project recently completed work to develop a substantive value framework for HTA in South Africa (SAVE-UHC, 2019) that could provide further grounding for the choice and design of HTA procedures.

## Conclusion

This study analysed landmark health rights cases in South Africa to inform the identification of country-specific procedural values related to health priority-setting and their implementation in HTA. Our findings indicate that A4R is likely to be insufficient for ensuring that HTA in South Africa meets the procedural demands of a constitutional rights-based approach to healthcare access. Accordingly, we suggest that a South African HTA body ought to consider more demanding considerations related to transparency and revisions as well as explicit considerations related to inclusivity.

To date, the transparent analysis of case law to inform the work of HTA bodies has been largely overlooked. Thus, an important contribution of the methodology described in this study is its potential application in national contexts other than South Africa, especially in countries where there is a constitutional basis for ensuring that all have access to healthcare or where judicialization of healthcare access occurs. Some of the findings reported here may also be directly transferable to other national contexts; for instance, any country where there is a commitment to progressively realize the right to health may want to ensure that revision is regularly undertaken as part of health priority-setting processes and not merely responsive to appeals.

## Supplementary Material

czab132_Supp

## Data Availability

The data underlying this article are available in the article and in its online supplementary material.

## Endnotes

1. ‘Social values’ as a concept is inconsistently defined and discussed in the literature. Some, like Clark and Weale, understand social values as contingent expressions of universal moral values and thus ‘justifiable’ through moral argument. A contrasting view, often found in the health economics literature, understands social values as descriptive statements of societal preferences that may or may not be morally justifiable. We adopt Clark and Weale’s conception given that the work described in this paper is intended to inform a larger project to develop a country-specific ‘ethics’ framework for HTA in South Africa. Ultimately, any insights drawn from case law that could not be morally justified would ideally be excluded from informing the work of this HTA body.
