# Supplementary material for: Integrating health technology assessment and the right to health: a qualitative content analysis of procedural values in South African judicial decisions
Source: Health Policy Plan. 2021 Nov 12;37(5):644–54. doi: 10.1093/heapol/czab132 (PMC9113169; doi:10.1093/heapol/czab132)
Supplement: czab132_Supp [file czab132_supp.zip › cross-ref approach.docx]

Details of cross-referencing approach to supplement case selection

1. Cases referenced in Cooper C. Health rights litigation: cautious constitutionalism. In: Yamin AE, Gloppen S, eds. *Litigating Health Rights: Can Courts Bring More Justice to Health?* Cambridge, MA: Harvard University Press; 2011:190-231.

- *Van Biljoen*
- *Soobramoney**
- *Grootboom*~
- *TAC**
- *Westville*
- *Mazibuko**
- *PMA*~
- *Hazel Tau*~
- *Affordable Medicines*~
- *New Clicks*
- *Goliath*~
- *TAC-Merck*
- *Woodcarb*~
- *Du Plooy*
- *Interim Procurement*~
- *Hichange*~
- *TAC-Rath*~
- *TAC-MM*~
- *Nokotyana*~

* indicates cases already selected for inclusion through systematic search (described in main text)

~ indicates decision was unrelated to section 27 or 35 according to Cooper’s analysis

1. Cases referenced in Bilchitz D. Health. In: Woolman S, Bishop M, eds. *Constitutional Law of* South Africa, Second Edition. Cape Town, South Africa: Juta & Company; 2014.
   - *Soobramoney**
   - *Grootboom*~
   - *TAC**
   - *Khosa**
   - *Van Biljoen*
   - *New Clicks*
   - *Ex Parte Chairperson of the Constitutional Assembly In Re: Certification of the Constitution of the Republic of South Africa*

* indicates cases already selected for inclusion through systematic search (described in main text)

~ indicates cases unrelated to section 27 or 35 according to Cooper’s analysis

1. Cases relating to the right to health care referenced in Currie I, De Waal J. *The Bill of Rights Handbook*. 6th ed. Cape Town, South Africa: Juta & Company; 2013: 563-597.
   - *Soobramoney**
   - *TAC**
   - *Grootboom*~
   - *Mazibuko**
   - *Van Biljoen*

* indicates cases already selected for inclusion through systematic search (described in main text)

~ indicates cases unrelated to section 27 or 35 according to Cooper’s analysis

1. The following cases relating to section 27 or 35 were identified by the three above references:

- ***Van Biljoen* (3x)**
  - Included due to reference by all 3 sources
- ***New Clicks* (2x)**
  - Included due to reference by 2 sources
- ***Westville***
  - Included on basis of expert judgment and to represent section 35
- *TAC-Merck*
  - Case withdrawn after companies acceded to TAC demands
- ***Du Plooy***
  - Included on basis of expert judgment and to represent section 35
- *Ex Parte Chairperson of the Constitutional Assembly In Re: Certification of the Constitution of the Republic of South Africa*
  - Excluded because this case addressed an update to the Constitution, not the interpretation of the rights therein

1. Four additional cases included:

*Van Biljoen, New Clicks, Westville, Du Plooy*
